# Immunogenicity and safety of co-administration with the Sabin-strain-based inactivated poliovirus vaccine (vero cell) and the diphtheria-tetanus-acellular pertussis vaccine in eligible children in China: a randomized, controlled, multicenter, non-inferiority trial

**DOI:** 10.3389/fimmu.2025.1633170

**Published:** 2025-09-03

**Authors:** Xiaoyu Liu, Shasha Han, Xiao Chen, Li Sun, Ruize Wang, Xuanwen Shi, Yu Guo, Hui Wang, Haiping Chen, Shaoying Chang, Xiaomeng Xu, Chao Zhang, Yinan Wang, Dan Zhang, Weijun Hu, Shaobai Zhang

**Affiliations:** ^1^ Immunization Program Division, Shaanxi Provincial Centre for Disease Control and Prevention, Xi’an, Shaanxi, China; ^2^ Medical Affairs Department, China National Biotec Group Company Limited, Beijing, China; ^3^ Immunization Program Division, Shanxi Provincial Center for Disease Control and Prevention, Taiyuan, China; ^4^ Department of Immunization Program Administration, Hebei Provincial Center for Disease Control and Prevention, Shijiazhuang, China; ^5^ Monitoring and Early Warning Division, Shaanxi Provincial Centre for Disease Control and Prevention, Xi'an, Shaanxi, China

**Keywords:** vaccine co-administration, DTaP, Sabin IPV, immunogenicity, safety

## Abstract

**Objective:**

In developing countries, combined vaccine availability remains limited due to economic constraints, healthcare infrastructure, and supply chain challenges. While some imported combined vaccines are available in China, their accessibility is restricted. Co-administration of individual vaccines presents a viable alternative. This study evaluates the immunogenicity and safety of simultaneous sIPV and DTaP administration to support vaccination policies and improve immunization rates.

**Methods:**

In this randomized, controlled, open-label, multicenter non-inferiority trial, 702 healthy 3-month-old infants from Shaanxi, Shanxi, and Hebei provinces were enrolled and assigned to three groups: Group 1 (sIPV + DTaP co-administration), Group 2 (sIPV alone), and Group 3 (DTaP alone). Vaccines were administered on a 3-4-5-month schedule. Serum samples were collected pre-vaccination and 30 days post-vaccination to assess antibody responses. Adverse events (AEs) were monitored for safety evaluation.

**Results:**

Among 671 infants completing the study (642 per protocol), co-administration (Group 1) demonstrated non-inferior immunogenicity compared to separate administration. Seroconversion rates and geometric mean titers (GMTs) for poliovirus types 1,2 and 3 were comparable between Groups 1 and 2. For anti-PT, FHA, D, T, Group 1 showed non-inferiority to Group 3 in seroconversion. However, anti-PT and anti-FHA geometric mean concentrations (GMCs) were lower (Group 1:anti-PT 31.06 [95% CI: 28.56–33.77], anti-FHA 29.40 [27.68–31.24]; Group 3: anti-PT 39.32 [36.25–42.65], anti-FHA 33.06 [31.01–35.24]). No significant differences were observed in anti-D and anti-T GMCs. AE rates were similar across groups, with local reactions (e.g., induration) more frequent in Group 1 (6.84%) than in Group 2 (0.85%). Systemic AEs (primarily grade 1–2 fever) did not differ significantly.

**Conclusion:**

Co-administration of sIPV and DTaP is immunogenically non-inferior to separate administration and demonstrates comparable safety. This strategy is feasible and may support simplified immunization schedules in China.

**Clinical trial registration:**

ClinicalTrials.gov, identifier NCT04053010.

## 1 Introduction

Vaccination represents the most effective preventive measure in clinical medicine, and the success of immunization programs relies on the efficacy and safety of vaccines and achieving high vaccine acceptance and coverage rates (1, 2). The immunization schedule in the Chinese vaccine program is intricate and involves multiple doses. With advancements in science and technology, novel non-immunization program vaccines are continually introduced to the market, leading to a continuous rise in vaccine doses administered to infants and young children. Co-administration reduces vaccine doses, lowers vaccination costs, saves healthcare resources, enhances vaccination rates, and strengthens immune protection (3–9). Simultaneously, in response to the global immunization strategy post-2020, the World Health Organization (WHO) has put forth high-level recommendations. These recommendations emphasize the need to strengthen country-led evidence-based decision-making, encourage countries to promote research to accelerate the uptake of vaccines and vaccine technologies, and improve program performance (10).

Globally, IPV schedules vary: The WHO recommends that all OPV-using countries adopt a schedule of 3 bOPV doses and 2 IPV doses. In endemic/high-risk regions, a bOPV birth dose (administered ≤1 week postpartum) is prioritized. The preferred schedule initiates bOPV at ≥6 weeks (4-week intervals) with IPV starting at ≥14 weeks (second dose ≥4 months later), maximizing immunogenicity. In countries with high vaccination coverage and low importation risk, an IPV-bOPV sequential schedule can be used. This regimen entails two initial IPV doses starting at ≥8 weeks of age (4–8 week interval), followed by ≥2 bOPV doses (4–8 week interval, adjusted to local exposure risk) In polio-free regions with very low importation risk and sustained high routine coverage (DTP3 > 90%), WHO considers an IPV-only schedule feasible but advises a gradual transition: first achieve high coverage with two IPV doses while maintaining bOPV use. Primary options include (1): 3-dose IPV series (6/8 weeks start, ≥4-week intervals; 6-week start mandates ≥6-month booster), or (2) 2-dose/fractional-dose schedule (≥14 weeks start, second dose ≥4 months later) (11). High-income countries demonstrate substantial heterogeneity in IPV schedules. In the United States, IPV is administered at 2, 4, and 6–18 months with a 4–6 year booster. In several European Union countries, the immunization schedules vary slightly. In France and Germany, the primary IPV series is delivered at 2, 4, and 11 months (France: boosters at 6 and 11–13 years; Germany: 9–16 years). Italy administers primary immunization at 2, 4, and 10 months with boosters at 5 and 12 years. In other countries such as Austria, Czechia, Denmark, and Finland, the first IPV dose is typically given at 3 months, with subsequent doses at 4–5 months and 11–13 months, depending on national schedules (12, 13). Likewise, DTaP schedules differ worldwide: WHO recommends a primary 3-dose series of DTP-containing vaccine, initiating as early as 6 weeks with ≥4-week intervals, and completion by 6 months when possible (14). The US employs a 5-dose DTaP schedule: primary doses at 2/4/6 months, boosters at 15–18 months and 4–6 years (12). These variations underscore the importance of evaluating co-administration strategies across diverse immunization programs, especially where scheduling overlap may occur.

According to the ‘National Immunization Program for Children (2016 Edition)’ (3) and vaccine instructions, the primary immunization for sIPV consists of three doses, with the initial dose administered at 2 months and subsequent doses given at at least 4–6 weeks intervals. For DTaP, the primary immunization involves three doses, starting at 3 months and concluding by 12 months, with each dose spaced at 4–6 week intervals. Suppose the sIPV vaccine is not administered with the 1^st^ dose during the initial immunization at 2 months; there may be a potential overlap in the subsequent vaccination schedule with DTaP. For the above reasons, and to alleviate the healthcare burden on parents and children associated with separate vaccine administration and reduce the risk of cross-infection among children in vaccination centers, we designed this study to compare the immunogenicity and safety of co-administration versus separate administration of sIPV and DTaP in eligible children.

## 2 Materials and methods

### 2.1 Study design

This randomized, controlled, open-label, multicenter design was conducted in Shaanxi, Shanxi, and Hebei provinces to evaluate the immunogenicity and safety of co-administration of sIPV and DTaP compared to separate administration. Ethical approval was obtained from the Ethics Committee of the Shaanxi Provincial Center for Disease Control and Prevention in June 2019 (Ethics Approval Number: SXSCDCIRB 2019-003). 702 healthy infants aged 3 months were recruited and randomly assigned to three groups: Group 1 received simultaneous administration of sIPV and DTaP, Group 2 received sIPV alone, and Group 3 received DTaP alone, each enrolled 234 participants. Vaccinations are scheduled at 3, 4, and 5 months. Vaccinations were administered by trained personnel after verifying recipient information to ensure accuracy. Each vaccine was given according to the dosage and administration site specified in the respective package inserts. sIPV was administered via intramuscular injection into the mid-anterolateral thigh, while DTaP was administered intramuscularly into the deltoid muscle of the upper arm. Regardless of the vaccine type, a standardized lateralization protocol was implemented: dose 1 - right side; dose 2 - left side; dose 3 - right side. If the designated injection site was deemed unsuitable (e.g., local injury), the side of administration (right/left) was adjusted accordingly, and the actual site was documented in the original case record. For the co-administration group, the two vaccines were injected into separate anatomical sites as described above. Additionally, we designed a catch-up vaccination schedule for participants in Group 2 and Group 3. For Group 2 participants, DTaP was administered 7–14 days after each sIPV vaccination at 3, 4, and 5 months of age. Conversely, for Group 3 participants, sIPV was administered 7–14 days after each DTaP vaccination at 3, 4, and 5 months of age. Blood samples were collected pre-vaccination and 30 days after post-vaccination for serum antibody level determination. Adverse events (AEs) following each vaccination were documented and analyzed to assess the immunogenicity and safety of co-vaccination compared to separate administration. The study was registered on ClinicalTrials.gov with the registration number NCT04053010.

### 2.2 Study subjects

The study population was 3 months or older on the day of enrollment who had not previously received sIPV, OPV, DTaP vaccines, or related combination vaccines. The participants’ legal guardians must sign and date the informed consent form and ≥14 days time interval since the last vaccination on the day of enrollment for participants. Before entering the study, a medical history review and clinical examination confirmed a body temperature of ≤37.0 °C. Exclusion criteria included: (1) a personal or family history of psychiatric illnesses; (2) undergoing treatment for malignancies, or experiencing immunosuppression due to HIV, or having family members with congenital immunodeficiency; (3) administration of non-specific immunoglobulin within the past month; (4) known or suspected concurrent diseases, including respiratory diseases, or acute infections; (5) various infectious, suppurative, and allergic skin diseases; (6) any conditions deemed by the investigator as potentially affecting the assessment of the trial.

### 2.3 Study vaccines

The sIPV (0.5 ml/dose, lot number 201901038, expiration date January 29, 2021) is produced by Beijing Institute of Biological Products Co., Ltd., contains 15, 45, and 45 D-antigen units (DU) of anti-polio types I, II, and III, respectively. The DTaP (0.5ml/dose, lot number 201809059-2, expiration date September 18, 2020) is manufactured by Wuhan Institute of Biological Products Co., Ltd., contained with not less than 4.0 international unit (IU) of acellular pertussis, not less than 30 IU of diphtheria, and not less than 40 IU of tetanus, respectively.

### 2.4 Stratified randomization and masking

This study is an open-label, multicenter clinical trial. Potential variations in subjects’ entry times were considered to ensure intergroup balance and enhance segment comparability. The 702 participants were divided into 78 block groups, and each block group consisted of 9 individuals with a stratified block randomization method. The statistical software SAS 9.4 was used for randomization to ensure equal and randomized distribution of subjects across Group 1, Group 2, and Group 3 throughout the trial. The Shaanxi Provincial Centers for Disease Control and Prevention provided a randomization list for subject allocation before three participating centers were enrolled. Information regarding group assignment was kept blind from investigators and the infants’ parents or legal guardians until after randomization. The specific vaccine administered to each subject was blinded to both the testing unit and during statistical analysis.

### 2.5 Immunogenicity assessment

The serum antibodies against the sIPV vaccine were detected using the cytopathic effect inhibition method (CPE) by the Chinese Center for Disease Control and Prevention (15). The test strains used were Sabin type I, type II, and type III, consistent with WHO-recommended strains for neutralization testing. Anti-polio types I, II, and III titers ≥1:8 were considered positive. Seroconversion was defined as follows: if pre-vaccination antibody titers were <1:8 and post-vaccination titers were ≥1:8, or if pre-vaccination titers were ≥1:8 and there was a fourfold or more significant increase in post-vaccination titers against polio types I, II, and III. The geometric mean titer (GMT) represented the serum antibody levels. Assuming the expected half-life of maternal antibodies was 28 days, the correction of the maternal antibody effect was calculated using the formula published by Luis Rivera (16). Immunoglobulin G (IgG) antibodies against DTaP were detected using enzyme-linked immunosorbent assay (ELISA) by China National Institutes for Food and Drug Control (NIFDC) to determine serum levels of pertussis toxoid (PT), filamentous hemagglutinin (FHA), tetanus (T), and diphtheria (D) pre and post-vaccination, expressed in IU/ml (17–20). Calibrations were performed against WHO International Standards: Anti-D and Anti-T antibodies used the WHO International Standard for Diphtheria Antitoxin (10/262) and Tetanus Antitoxin (TE-3), respectively; Anti-PT and Anti-FHA antibodies were quantified with Human Pertussis Antiserum International Reference Standard 06/140. Anti-D and Anti-T ≥0.1 IU/ml, and Anti-PT and Anti-FHA ≥20 IU/ml were considered positive. Seroconversion was defined as pre-vaccination Anti-D and Anti-T <0.1 IU/ml, with post-vaccination levels ≥0.1 IU/ml, or pre-vaccination Anti-PT and Anti-FHA <20 IU/ml, with post-vaccination levels ≥20 IU/ml. Alternatively, individuals with positive pre-vaccination titers and a fourfold or more significant increase in post-vaccination titers were considered seroconversion.

### 2.6 Safety assessment

All participants were observed for 30 minutes after each dose by the investigators to monitor any adverse events (AEs). The subjects’ guardians recorded adverse events (AEs) occurring within 7 days and 8–30 days post-vaccination using diary cards. Meanwhile, investigators collect data on serious adverse events (SAEs) occurring within 3 months after the final immunization dose via telephone follow-ups. Local solicited AEs, such as pain, induration, redness, swelling, rash, skin and mucous membranes, and systemic solicited AEs, like fever, irritability, vomiting, diarrhea, somnolence, eating disorder, and allergic reactions, were recorded. All AEs were classified and assessed in correlation with the vaccine according to the Guiding Principles for Grading Adverse Reactions in Clinical Trials of Preventive Vaccines issued by the National Medical Products Administration (21).

### 2.7 Data analysis

Statistical analysis was conducted using SAS (r) Proprietary Software 9.4 (TS1M7). Safety and immunogenicity analyses were descriptive and utilized Pearson’s chi-square test or Fisher’s exact test, as well as non-parametric tests for data analysis. Seroconversion rates and geometric mean titers/concentrations (GMT/GMC) for each vaccine were calculated along with their respective 95% confidence intervals (CIs). Analysis of variance was employed to compare the GMT/GMC between groups after logarithmic transformation. Statistical significance was determined at *P*< 0.05. Non-inferiority criteria were established as follows: the lower limit of the 95% CI for the difference in seroconversion rates between the study group and the control group was set at ≥ -10% and the lower limit of the 95% CI for the GMT/GMC ratio between the study group and the control group was set at ≥ 0.67.

## 3 Results

### 3.1 Baseline demographic characteristics

**Figure 1:**
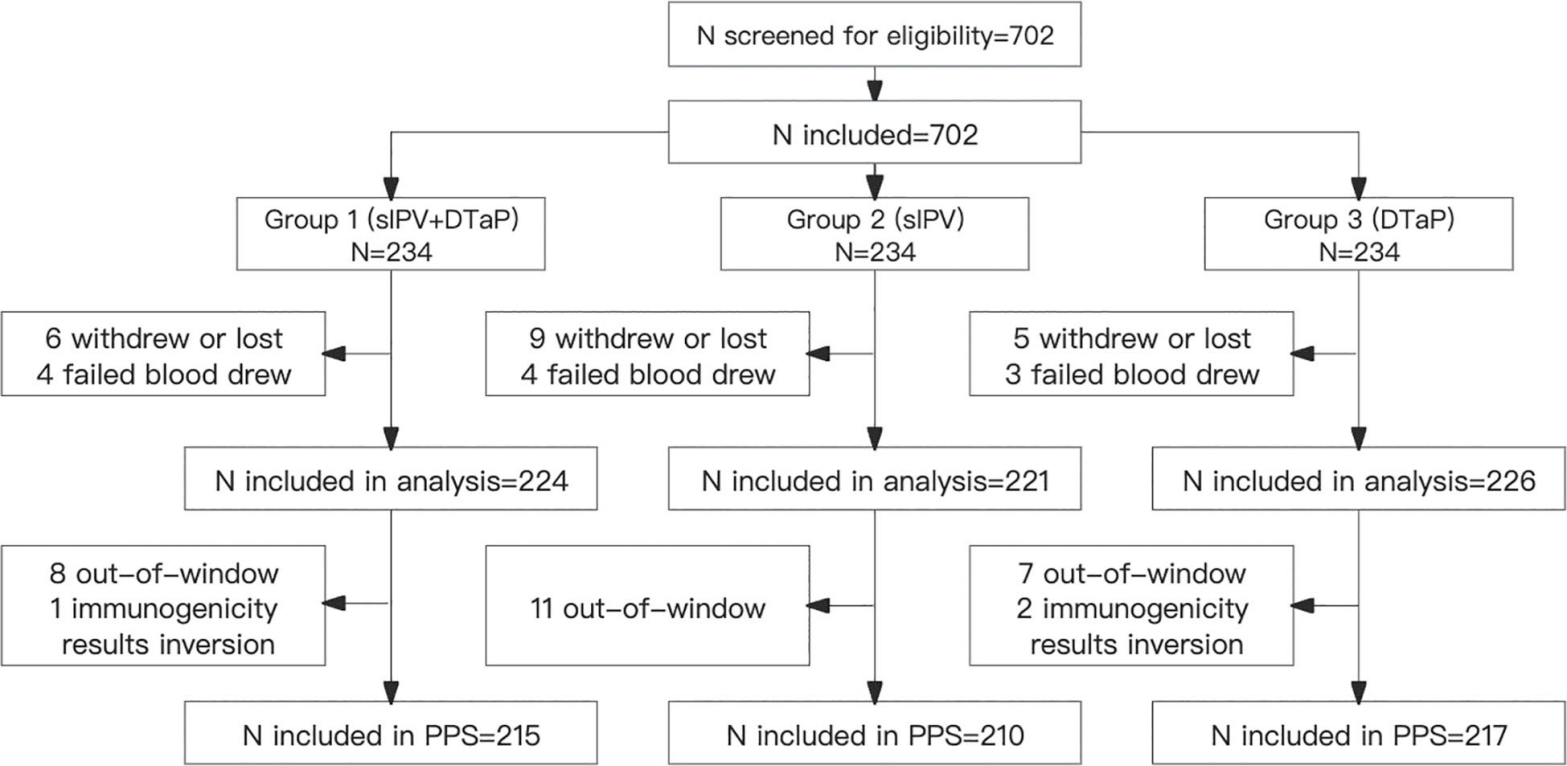
Trial flow chart, subject disposition.

A total of 702 participants were randomized in a 1:1:1 ratio into Group 1 (sIPV and DTaP co-administration), Group 2 (sIPV administration), and Group 3 (DTaP administration) during the trial. Thirty-one participants dropped out: 10 from Group 1, 13 from Group 2, and 8 from Group 3, respectively. During the study period, there were 29 protocol deviations: 9 in Group 1, 11 in Group 2, and 9 in Group 3, respectively. Eventually, 642 subjects were included in the per-protocol population. Baseline demographic characteristics were comparable across all groups (**Table 1**, **Figure 1**).

**Table 1:** Baseline characteristics, PPS.

Characteristic	Group 1, N=215	Group 2, N=210	Group 3, N=217	*P* value
Age (day),D (IQR)	100 (95,107)	101 (96,109)	101 (96,108)	0.148
Gender				0.868
Male, n (%)	111 (51.63)	104 (49.52)	107 (49.31)	
Female, n (%)	104 (48.37)	106 (50.48)	110 (50.69)	
Body weight (kg),D (IQR)	7 (6.5,7.6)	7 (6.4,7.6)	7 (6.4,7.6)	0.759
Body height (cm),D (IQR)	63 (61,65)	63 (61,65)	63 (61,65)	0.972

### 3.2 Immunogenicity

**Figure 2:**
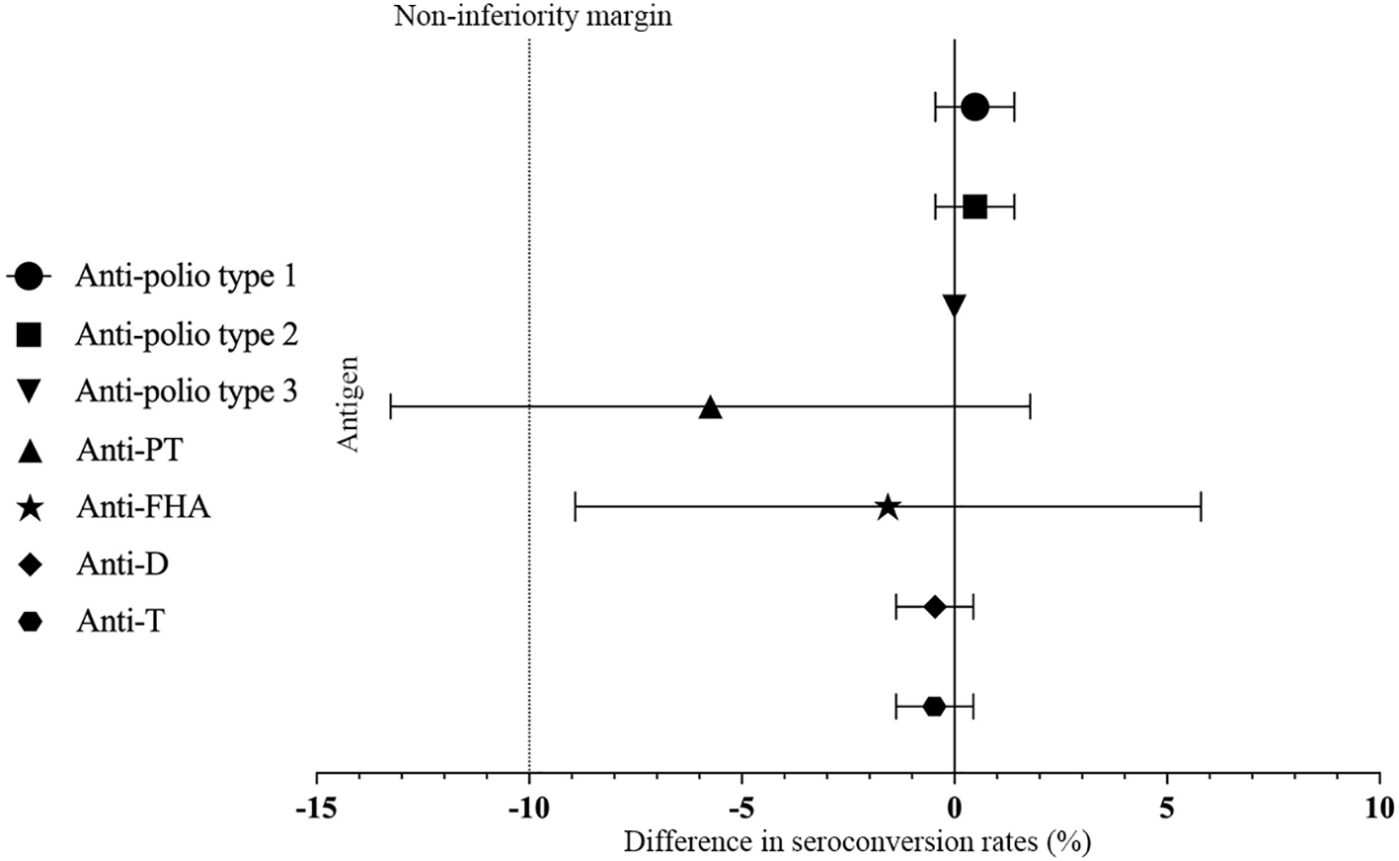
Differences rate in the proportion of seroconversion. PT, pertussis toxoid; FHA, filamentous hemagglutinin; D, Diphtheria; T, Tetanus. Differences in the proportion of seroconversion to anti-polio type 1, 2, and 3 were measured between Group 1 and Group 2 with two-sided 95% CIs, and differences in the proportion of seroconversion to anti-PT, anti-FHA, anti-D and anti-T were measured between Group 1 and Group 3 with two-sided 95% CIs.

**Figure 3:**
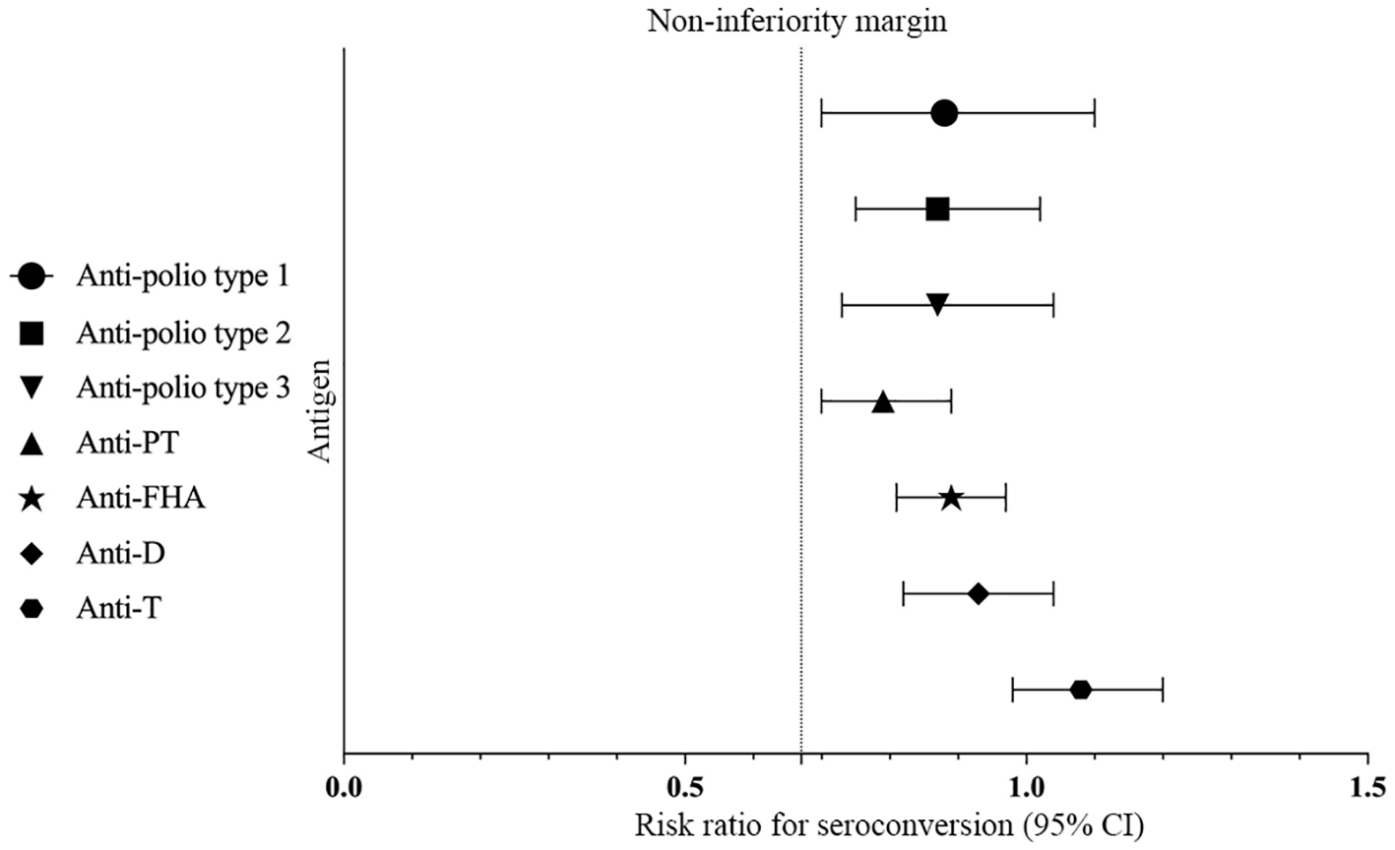
Differences in ratios of GMTs/GMCs. Differences in ratios of GMTs to anti-polio types 1, 2, and 3 were measured between Group 1 and Group 2 with two-sided 95% CIs, and differences in ratios of GMCs to anti-PT, anti-FHA, anti-D, and anti-T were measured between Group 1 and Group 3 with two-sided 95% CIs.

The seropositive rates of anti-polio types I, II, and III did not show a statistically significant difference between Group 1 and Group 2 (*P* > 0.05) pre-vaccination. Similarly, there were no statistically significant differences in the seropositive rates and seroconversion rates of all three types between Group 1 and Group 2 (irrespective of the correction for maternal antibodies) (*P*>0.05) post-vaccination. Additionally, the seroconversion rates for anti-polio types I, II, and III in Group 1 were non-inferior to those in Group 2 (lower limit of the 95% CI for the difference in seroconversion rates between Group 1 and Group 2 ≥ -10%). The post-vaccination geometric mean titers (GMTs) for anti-polio types I, II, and III in Group 1 were 1015.73 (95% CI: 875.15-1178.90), 294.54 (95% CI: 266.32-325.75), and 580.60 (95% CI: 514.45-655.27), respectively. In Group 2, the corresponding GMTs were 1157.11 (95% CI: 977.99-1369.04), 337.06 (95% CI: 300.82-377.68), and 665.64 (95% CI: 587.13-754.65), with no statistically significant differences observed between the two groups (*P* > 0.05). Furthermore, the GMTs for all three types in Group 1 were non-inferior to those in Group 2 (lower limit of the 95% CI for the GMT ratio between Group 1 and Group 2 ≥ 0.67) (**Table 2**, **Figure 2**, **Figure 3**).

**Table 2:** Seropositive/seroconversion rates and GMTs 30 days after the 3rd dose between Group1 and Group2, PPS.

Serum antibodies	Parameters of immunogenicity		Group 1, N=215	Group 2, N=210	*P* value
Anti-type I	Seropositive, Rate% [95% CI]	Pre-1^st^ dose	20 [14.65, 25.35]	20.95 [15.45, 26.45]	0.808
		Post-3^rd^ dose	100 [98.30, 100]	100 [98.26, 100]	1.000
	Seroconversion, Rate% [95% CI]	Post-3^rd^ dose,Unmodified maternal antibodies	99.07 [97.79, 100]	96.67 [94.24, 99.10]	0.077
		Post-3^rd^ dose,Modified maternal antibodies	100 [98.30,100]	99.52 [98.59,100]	1.000
	GMT [95% CI]	Pre-1^st^ dose	5.00 [4.68, 5.34]	5.44 [4.94, 5.99]	0.638
		Post-3^rd^ dose	1015.73 [875.15,1178.90]	1157.11 [977.99, 1369.04]	0.062
Anti-type II	Seropositive, Rate% [95% CI]	Pre-1^st^ dose	9.30 [5.78, 14.00]	11.90 [7.85, 17.07]	0.383
		Post-3^rd^ dose	100 [98.30, 100]	99.52 [97.38, 99.99]	0.494
	Seroconversion, Rate% [95% CI]	Post-3^rd^ dose,Unmodified maternal antibodies	99.53 [97.44, 99.99]	99.52 [97.38, 99.99]	1.000
		Post-3^rd^ dose,Modified maternal antibodies	100 [98.30, 100]	99.52 [97.38, 99.99]	0.494
	GMT [95% CI]	Pre-1^st^ dose	4.43 [4.24, 4.64]	4.56 [4.32, 4.82]	0.413
		Post-3^rd^ dose	294.54 [266.32, 325.75]	337.06 [300.82, 377.68]	0.095
Anti-type III	Seropositive, Rate% [95% CI]	Pre-1^st^ dose	8.37 [4.67, 12.07]	4.76 [1.88, 7.64]	0.134
		Post-3^rd^ dose	100 [98.30, 100]	100 [98.26, 100]	1.000
	Seroconversion, Rate% [95% CI]	Post-3^rd^ dose,Unmodified maternal antibodies	98.14 [96.33, 99.95]	100 [98.260, 100]	0.129
		Post-3^rd^ dose,Modified maternal antibodies	100 [98.30, 100]	100 [98.26, 100]	1.000
	GMT [95% CI]	Pre-1^st^ dose	4.63 [4.28, 5.01]	4.29 [4.09, 4.50]	0.130
		Post-3^rd^ dose	580.6 [514.45, 655.27]	665.64 [587.13, 754.65]	0.131

There was no statistically significant difference in the post-vaccination seroconversion rates for anti-PT, anti-FHA, anti-D, and anti-T between Group 1 and Group 3 (*P* > 0.05). Additionally, the post-vaccination seroconversion rates for anti-FHA, anti-D, and anti-T in Group 1 were non-inferior to Group 3 (with the lower limit of the 95% CI for the difference in seroconversion rates between the study group and control group two being ≥ -10%). The post-vaccination geometric mean concentrations (GMC) for anti-D were 1.50 (95% CI: 1.37-1.63) in Group 1 vs. 1.61 (95% CI: 1.49-1.75) in Group 3, and for anti-T, they were 4.62 (95% CI: 4.27-4.99) in the Group 1 vs. 4.26 (95% CI: 4.01-4.54) in Group 3. The differences between the two groups were not statistically significant (*P* > 0.05), and the post-vaccination ratios of anti-D and anti-T GMC in Group 1 to Group 3 were 0.93 (0.82, 1.04) and 1.08 (0.98, 1.20), respectively, indicating non-inferiority for the two groups (with the lower limit of the 95% CI for the ratio being ≥ 0.67). Although the post-vaccination GMC for anti-PT (31.06 vs. 39.32, *P* < 0.001) and anti-FHA (29.40 vs. 33.06, *P* = 0.009) in Group 1 were slightly lower than in Group 3, the post-vaccination GMCs for anti-PT and anti-FHA in Group 1 were non-inferior to Group 3 (with the lower limit of the 95% CI for the GMT ratio between the study group and control group one being ≥ 0.67) (**Table 3**, **Figure 2**, **Figure 3**).

**Table 3:** Seropositive/seroconversion rates and GMCs 30 days after the 3rd dose between Group1 and Group3, PPS.

Serum antibodies	Parameters of immunogenicity		Group 1, N=215	Group 3, N=217	*P* value
anti-PT	Seropositive, Rate% [95% CI]	Pre-1^st^ dose	0.47 [0.01, 2.56]	1.84 [0.50, 4.65]	0.166
		Post-3^rd^ dose	77.67 [71.51, 83.06]	84.33 [78.80, 88.90]	0.078
	Seroconversion, Rate% [95% CI]	Post-3^rd^ dose	77.21 [71.01, 82.64]	82.95 [77.27, 87.70]	0.135
	GMC [95% CI]	Pre-1^st^ dose	2.25 [2.06, 2.46]	2.49 [2.24, 2.76]	0.243
		Post-3^rd^ dose	31.06 [28.56, 33.77]	39.32 [36.25, 42.65]	<0.001
anti-FHA	Seropositive, Rate% [95% CI]	Pre-1^st^ dose	0.47 [0.01, 2.56]	4.15 [1.91, 7.73]	0.006
		Post-3^rd^ dose	80.93 [75.03, 85.95]	86.18 [80.86, 90.47]	0.141
	Seroconversion, Rate% [95% CI]	Post-3^rd^ dose	80.47 [74.53, 85.54]	82.03 [76.26, 86.90]	0.677
	GMC [95% CI]	Pre-1^st^ dose	3.03 [2.70, 3.40]	3.42 [3.02, 3.88]	0.163
		Post-3^rd^ dose	29.40 [27.68, 31.24]	33.06 [31.01, 35.24]	0.009
anti-D	Seropositive, Rate% [95% CI]	Pre-1^st^ dose	2.79 [1.03, 5.97]	1.84 [0.50, 4.65]	0.513
		Post-3^rd^ dose	100 [98.30, 100]	100 [98.31, 100]	1.000
	Seroconversion, Rate% [95% CI]	Post-3^rd^ dose	99.53 [97.44, 99.99]	100 [98.31, 100]	0.498
	GMC [95% CI]	Pre-1^st^ dose	0.01 [0.01, 0.01]	0.02 [0.02, 0.02]	<0.001
		Post-3^rd^ dose	1.50 [1.37, 1.63]	1.61 [1.49, 1.75]	0.211
anti-T	Seropositive, Rate% [95% CI]	Pre-1^st^ dose	5.12 [2.85, 9.01]	2.30 [0.96, 5.42]	0.122
		Post-3^rd^ dose	100 [98.30, 100]	100 [98.31, 100]	1.000
	Seroconversion, Rate% [95% CI]	Post-3^rd^ dose	99.53 [97.44, 99.99]	100 [98.31, 100]	0.498
	GMC [95% CI]	Pre-1^st^ dose	0.02 [0.02, 0.02]	0.02 [0.02, 0.02]	0.282
		Post-3^rd^ dose	4.62 [4.27, 4.99]	4.26 [4.01, 4.54]	0.116

### 3.3 Safety

**Figure 4:**
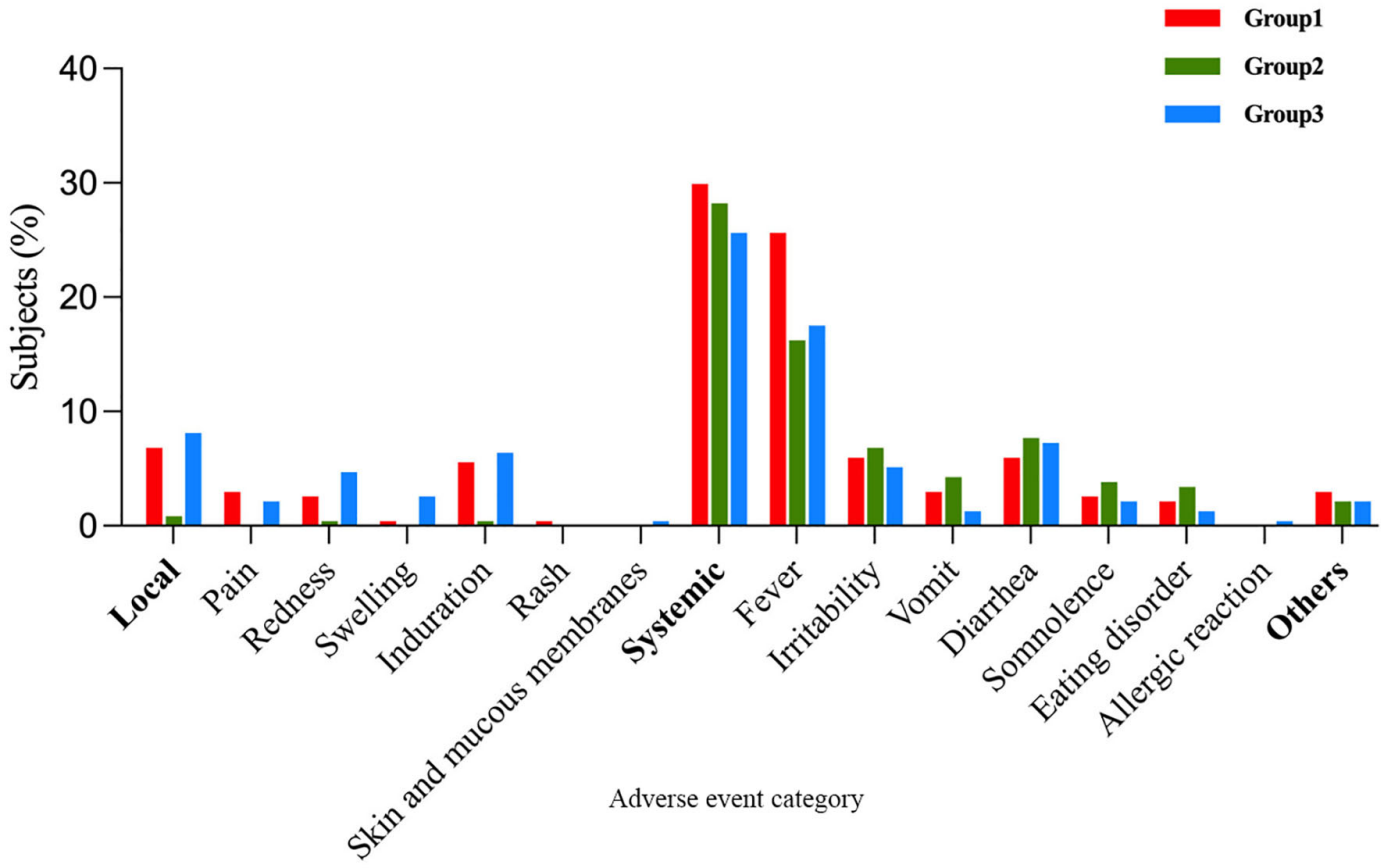
Vaccine-related local and systemic AEs within 7 days after any dose.

Within 7 days post-vaccination, there were 77 (32.91%), 67(28.63%), and 74 (31.62%) vaccine-related AEs occurrences in Group 1, Group 2, and Group, respectively. The differences in AE incidence rates among the three groups were not statistically significant (Group 1 vs. Group 2, *P*=0.317; Group 1 vs. Group 3, *P* = 0.767) (**Table 4**). When comparing Group 1 with Group 2, there was no statistically significant difference in the incidence rates of vaccine-related systemic AEs and non-solicited AEs (*P* = 0.684; *P* = 0.559), but the incidence of local AEs in Group 1 was higher than in Group 2 (6.84% vs. 0.85%; *P*=0.001). Further comparison revealed that the incidence rates of vaccine-related pain, redness, induration, and fever in Group 1 were all higher than in Group 2 (pain: 2.99% vs. 0%, *P*=0.002; redness: 2.56% vs. 0.43%, *P*=0.045; induration: 5.56% vs. 0.43%, *P*=0.001; fever: 25.64% vs. 16.24%, *P*=0.012). Additionally, no statistically significant differences existed in the incidence rates of other vaccine-related AEs’ symptoms. When comparing Group 1 with Group 3, except for the incidence rate of vaccine-related swelling being lower in Group 1 than in Group 3 (0.43% vs. 2.56%; *P* = 0.045), and the incidence rate of fever being higher in Group 1 than in Group 3 (25.64% vs. 17.52%; *P* = 0.033), there were no statistically significant differences in the incidence rates of other vaccine-related AEs’ symptoms (**Table 5**). Induration and fever were the most common local and systemic AE, respectively (**Table 5**, **Figure 4**). Except for the incidence rate of vaccine-related mild local AEs being higher in Group 1 than in Group 2 (6.41% vs. 0.43%; *P*<0.001), there were no significant differences in the severity of AEs among the groups, with most events being mild to moderate (**Table 6**). Similar results were observed for vaccine-related AEs occurring within 30 days post-vaccination. During the study period, 16 serious adverse events (SAEs) were reported (2.28%), all hospitalized cases. Only 1 case (0.14%) in Group 2 (sIPV) was possibly vaccine-related, presenting acute urticaria symptoms on the second day post-vaccination and recovering after hospitalization.

**Table 4:** Overall adverse events (AEs) in two groups, SS.

Correlation	Group 1, N=234		Group 2, N=234		*P* value	Group 3, N=234		*P* value
	n	Rate%	n	Rate%		n	Rate%	
Vaccine-related	77	32.91	68	29.06	0.368	75	32.05	0.844
Overall	77	32.91	67	28.63	0.317	74	31.62	0.767

**Table 5:** Local and systemic AEs within 7 days after any dose, SS.

AEs	Vaccine-related					Overall				
	Group1 (N=234)	Group2 (N=234)	*P* value	Group3 (N=234)	*P* value	Group1 (N=234)	Group2 (N=234)	*P* value	Group3 (N=234)	*P* value
**Local, n (Rate%)**	16 (6.84)	2 (0.85)	0.001	19 (8.12)	0.598	16 (6.84)	2 (0.85)	0.001	20 (8.55)	0.488
Pain, n (Rate%)	7 (2.99)	0 (0)	0.002	5 (2.14)	0.559	7 (2.99)	0 (0)	0.002	5 (2.14)	0.559
Redness, n (Rate%)	6 (2.56)	1 (0.43)	0.045	11 (4.70)	0.217	6 (2.56)	1 (0.43)	0.045	11 (4.70)	0.217
Swelling, n (Rate%)	1 (0.43)	0 (0)	1.000	6 (2.56)	0.045	1 (0.43)	0 (0)	1.000	6 (2.56)	0.045
Induration, n (Rate%)	13 (5.56)	1 (0.43)	0.001	15 (6.41)	0.697	13 (5.56)	1 (0.43)	0.001	16 (6.84)	0.565
Rash, n (Rate%)	1 (0.43)	0 (0)	1.000	0 (0)	1.000	1 (0.43)	0 (0)	1.000	0 (0)	1.000
Skin and mucous membranes, n (Rate%)	0 (0)	0 (0)	1.000	1 (0.43)	1.000	0 (0)	0 (0)	1.000	1 (0.43)	1.000
**Systemic, n (Rate%)**	70 (29.91)	66 (28.21)	0.684	60 (25.64)	0.302	77 (32.91)	76 (32.48)	0.922	72 (30.77)	0.620
Fever, n (Rate%)	60 (25.64)	38 (16.24)	0.012	41 (17.52)	0.033	68 (29.06)	48 (20.51)	0.032	50 (21.37)	0.055
Irritability, n (Rate%)	14 (5.98)	16 (6.84)	0.706	12 (5.13)	0.687	15 (6.41)	16 (6.84)	0.853	12 (5.13)	0.552
Vomit, n (Rate%)	7 (2.99)	10 (4.27)	0.459	3 (1.28)	0.201	9 (3.85)	11 (4.70)	0.648	3 (1.28)	0.079
Diarrhea, n (Rate%)	14 (5.98)	18 (7.69)	0.464	17 (7.26)	0.577	17 (7.26)	21 (8.97)	0.498	22 (9.40)	0.403
Somnolence, n (Rate%)	6 (2.56)	9 (3.85)	0.431	5 (2.14)	0.760	8 (3.42)	11 (4.70)	0.482	6 (2.56)	0.587
Eating disorder, n (Rate%)	5 (2.14)	8 (3.42)	0.399	3 (1.28)	0.473	6 (2.56)	9 (3.85)	0.431	5 (2.14)	0.760
Allergic reaction, n (Rate%)	0 (0)	0 (0)	1.000	1 (0.43)	1.000	0 (0)	0 (0)	1.000	1 (0.43)	1.000
**Others, n (Rate%)**	7 (2.99)	5 (2.14)	0.559	5 (2.14)	0.559	21 (8.97)	24 (10.26)	0.638	27 (11.54)	0.361

Local: Local adverse events; Systemic: Systemic adverse events; Others: Non-solicited adverse events; n: Number of participants experiencing adverse events.

**Table 6:** Severity of AEs within 7 days after any dose, SS.

AEs	Vaccine-related								Overall		
	Group 1 (N=234)	Group 2 (N=234)	*P* value	Group 3 (N=234)	*P* value	Group 1 (N=234)	Group 2 (N=234)	*P* value		Group 3 (N=234)	*P* value
All											
Mild, n (Rate%)	60 (25.64)	48 (20.51)	0.188	60 (25.64)	1.000	75 (32.05)	69 (29.49)	0.548		81 (34.62)	0.556
Moderate, n (Rate%)	40 (17.09)	37 (15.81)	0.708	34 (14.53)	0.447	43 (18.38)	39 (16.67)	0.627		38 (16.24)	0.541
Severe, n (Rate%)	4 (1.71)	7 (2.99)	0.360	5 (2.14)	0.736	5 (2.14)	7 (2.99)	0.559		5 (2.14)	1.000
Local											
Mild, n (Rate%)	15 (6.41)	1 (0.43)	<0.001	18 (7.69)	0.588	15 (6.41)	1 (0.43)	<0.001		19 (8.12)	0.476
Moderate, n (Rate%)	3 (1.28)	1 (0.43)	0.304	5 (2.14)	0.473	3 (1.28)	1 (0.43)	0.304		5 (2.14)	0.473
Severe, n (Rate%)	0 (0)	0 (0)	1.000	1 (0.43)	1.000	0 (0)	0 (0)	1.000		1 (0.43)	1.000
Systemic											
Mild, n (Rate%)	53 (22.65)	43 (18.38)	0.252	45 (19.23)	0.363	62 (26.50)	54 (23.08)	0.392		56 (23.93)	0.523
Moderate, n (Rate%)	36 (15.38)	34 (14.53)	0.795	26 (11.11)	0.173	38 (16.24)	36 (15.38)	0.800		29 (12.39)	0.235
Severe, n (Rate%)	0 (0)	0 (0)	0.360	1 (0.43)	1.000	5 (2.14)	7 (2.99)	0.559		4 (1.71)	0.736

## 4 Discussion

Eligible children should receive vaccinations according to national immunization schedules and guidelines to prevent common infectious diseases. In China, children must receive 19 doses of routine immunization vaccines before age 3. If all non-routine immunization vaccines are also chosen, 17 doses are required. In many cases, the timing of these vaccine administrations overlaps (8). Lowering the cost of implementing expanded immunization programs while ensuring vaccine efficacy has become an essential issue in public health.

Previous studies have indicated that co-administration of sIPV and DTaP at 3 and 4 months does not interfere with antibody seroconversion, and the high seroconversion rates and antibody titers observed in the co-administration group ensure infants are protected against these diseases (22). In this study, we aim to investigate further the impact of simultaneous administration of sIPV and DTaP at 3, 4, and 5 months of age on children’s immune response.

The study results demonstrate that co-administration of sIPV and DTaP compared to separate administration of the two vaccines yields non-inferior immune responses for all types of serum antibody GMT/GMC, with no statistically significant differences in seroconversion rates. These data support the strategy of sIPV and DTaP co-administration into routine childhood immunization programs. Despite the approval of the DTaP-IPV-Hib vaccine in China in May 2011, widespread use is still limited in most low- to middle-income families and resource-poor areas due to cost considerations. Immunization with routine scheduled vaccines remains the preferred option in these households and regions. Multiple studies have shown that the levels of various antibodies and seroconversion rates are similar when administering imported DTaP-IPV-Hib or DTaP-IPV vaccines compared to separate administration of monovalent vaccines (23–27). A phase III clinical trial conducted in China compared the immunogenicity and safety of the imported DTaP-IPV//PRPT combined vaccine to the co-administration of DTaP, Hib conjugate vaccine, and IPV at different injection sites, all administered at 3, 4, and 5 months. The results showed non-inferiority for each antigen’s serum protection rate/seroconversion rate. The serum detection methods used in the study were consistent with those used in this research. Even the absolute values of antibodies against poliovirus types 1, 2, and 3, diphtheria antibodies, and tetanus antibodies GMT/GMI were lower than those in this study (23), further confirming the feasibility of DTaP and sIPV co-administration to achieve similar infectious disease prevention as imported combination vaccines. Our study also provides robust data support for implementing evidence-based vaccination strategies.

A prospective observational cohort study compared the safety of routine childhood vaccinations administered simultaneously versus separately in the real-world setting from 2008 to 2018 in the United Kingdom, and the study found that the co-administration of two vaccines had similar or better safety profiles compared to separate administration of each vaccine (28). Our study results indicated no significant difference in the occurrence of adverse reactions within 7 days between the co-administration group and the separate administration group. Injection site induration and fever were the most common local and systemic symptoms, with a significantly higher induration rate in Group 1 (5.56%) compared to Group 2 (0.43%), with no significant difference from Group 3 (6.41%). The fever occurrence rate in Group 1 (25.64%) was higher than in Group 2 (16.24%) and Group 3 (17.52%), consistent with our previous findings (22). Additionally, the pain and redness occurrence rates in Group 1 (2.99%; 2.56%) were significantly higher than in Group 2 (0%; 0.43%), with no significant difference from Group 3 (2.14%; 4.70%). The higher local reaction rates in Group 1 (6.84%) and Group 3 (8.12%) may be attributed to standard vaccine components present in DTaP, such as pertussis toxin, diphtheria toxin, tetanus toxin, and aluminum (29). This is consistent with the findings of a safety surveillance study conducted in China, in which the local reaction rate was 8.56% after the third dose of the same DTaP vaccine (30). Most adverse events were mild to moderate in severity, and the prognosis was favorable.

During the study period, 16 serious adverse events (SAEs) occurred, which included 11 acute bronchitis cases(68.75%), 2 acute upper respiratory tract infection cases (12.50%), 2 acute urticaria case (12.50%), and 1 herpetic pharyngitis case (6.25%). Acute bronchitis often occurs during the cold season or sudden temperature drops (31, 32), coinciding with the period of clinical research conducted from August 2019 to January 2020, which aligned with China’s severe winter. Additionally, acute bronchitis is also the most common cause of hospitalization among infants during their first 12 months of life (33). These may have contributed to the higher hospitalization rate among the study participants. In Group 2 (sIPV), 1 SAE case was possibly related, where acute urticaria symptoms appeared on the second day after the first dose of sIPV vaccination. Acute urticaria is a potential adverse reaction mentioned in the sIPV vaccine package insert and may indicate an allergic reaction to any vaccine component. The child recovered after hospital treatment. The DTaP vaccine from the Wuhan Institute of Biological Products has been used in China for decades. At the same time, the sIPV from the Beijing Institute of Biological Products was licensed in 2017 (34) and prequalified by the World Health Organization (WHO) in 2022. Both vaccines have demonstrated exemplary safety profiles in practical use.

Although several clinical studies on the co-administration of multiple vaccines were constructed in China, this study is the first clinical study of the co-administration of sIPV and DTaP at 3, 4, and 5 months. Our study data can further support the safety of co-administration in Chinese and even Asian children, reducing vaccine hesitancy (35) among parents concerned about the safety of concurrent vaccine use.

Moreover, China’s national DTaP immunization schedule was updated on January 1, 2025, to doses at 2, 4, 6, and 18 months (with a booster at 6 years), replacing the earlier 3/4/5-month regimen (36). Although our study employed the former schedule, both approaches aim to confer early and robust pertussis protection. Published data demonstrate comparable immunogenicity between the 3/4/5 and 2/4/6 month schedules, while initiating at 2 months may further lower pertussis incidence, underscoring the continued relevance of our co-administration results (37). Future research can assess sIPV + DTaP co-administration under the current 2/4/6 month framework to confirm feasibility and effectiveness within the revised policy. On the other hand, despite the routine sIPV schedule (2, 3, and 4 months) recommended by China’s National Immunization Program, a randomized, controlled, non-inferiority trial conducted by our group demonstrated that co-administration of sIPV and DTaP at 2, 3, and 4 months achieved seroconversion rates of ≥95.29% for poliovirus types I–III and GMTs ranging from 153.43 to 369.00 at 30 days post-primary series (22). In the present study, despite using a later schedule (3, 4, and 5 months), we observed similarly high seroconversion rates (≥98.14%) and GMTs (294.54–1015.73) across all antigens. These findings are consistent with published data showing comparable immune responses whether sIPV and DTaP are administered at 2–4 months or 3–5 months, thereby reinforcing the operational flexibility of co-administration strategies within routine immunization programs.

This study has some limitations. Our extensive exclusion criteria—while enhancing internal validity—may limit generalizability to the broader pediatric population, as infants with specific comorbidities were not represented. Future studies should consider more inclusive enrollment to assess real-world applicability. Although seroconversion rates for all DTaP antigens met non-inferiority criteria, the observed lower GMCs of anti-PT and anti-FHA antibodies in the co-administration group compared to the DTaP-alone group warrant consideration. While these differences did not compromise short-term seroprotection, they may potentially influence the durability of immunity against pertussis—a concern heightened by China’s rising pertussis incidence (38). Future studies should include longer-term follow-up to assess antibody persistence and clinical protection, as well as evaluate whether supplemental booster doses can optimize long-term immunity within co-administration schedules. Additionally, although laboratory assays and data analyses were appropriately blinded, the open-label design could have introduced performance and observer bias, particularly in the reporting of subjective adverse events by caregivers and investigators. These methodological constraints should be considered when interpreting our safety outcomes.

The study indicates that co-administration is an effective way to improve vaccine coverage rates. During this study, the sudden onset of the COVID-19 pandemic in December 2019 highlighted the importance of timely immunization for the population during a significant infectious disease outbreak (39). Our research demonstrates that co-administration of sIPV and DTaP does not compromise immunogenicity and exhibits good safety profiles compared to separate administration. In public health strategies, the practice of co-administration is crucial for optimizing vaccination schedules, introducing new vaccines into immunization programs, and increasing coverage rates, which is cost-effective (40, 41). This is particularly significant given the increasing number of reported pertussis cases in China in recent years, emphasizing the crucial value of timely vaccination (42). Additionally, to evaluate the durability of the immune responses observed in this trial, we have initiated a follow-up study in the same cohort of 18-month-old children who received co-administration of sIPV, DTaP, and HepA-L. A five-year persistence protocol is also under development, which will include serial serologic assessments to characterize antibody kinetics over time. These data will be essential for informing optimal booster schedules within the national immunization program.

## Data Availability

The raw data supporting the conclusions of this article will be made available by the authors, without undue reservation.
