# Supplementary material for: Immunogenicity and safety of co-administration with the Sabin-strain-based inactivated poliovirus vaccine (vero cell) and the diphtheria-tetanus-acellular pertussis vaccine in eligible children in China: a randomized, controlled, multicenter, non-inferiority trial
Source: Front Immunol. 2025 Sep 3;16:1633170. doi: 10.3389/fimmu.2025.1633170 (PMC12440916; doi:10.3389/fimmu.2025.1633170)
Supplement: Supplementary file 1 [file SupplementaryFile1.docx]

**Supplementary Tables**

**Supplementary Table 1** Seroconversion rates and GMTs 30 days after the 3rd dose in susceptible and non-susceptible infants between Group 1 and Group 2, PPS.

| **Serum antibodies** | **Parameters of immunogenicity** | **Group 1** | **Group 2** | ***P* value** |
| --- | --- | --- | --- | --- |
| **Susceptible** |  |  |  |  |
| Anti-type I | n | 172 | 166 |  |
|  | Seroconversion, Rate% [95% CI] | 100[97.88, 100] | 100[97.80, 100] | 1.000 |
|  | GMT [95% CI] | 1153.22[984.50, 1350.85] | 1374.44[1162.71, 1624.73] | 0.073 |
| Anti-type II | n | 195 | 185 |  |
|  | Seroconversion, Rate% [95% CI] | 100[98.13, 100] | 99.46[97.03, 99.99] | 0.487 |
|  | GMT [95% CI] | 305.79[276.52, 338.17] | 340.14[302.2, 382.84] | 0.133 |
| Anti-type III | n | 197 | 200 |  |
|  | Seroconversion, Rate% [95% CI] | 100[98.14, 100] | 100[98.17, 100] | 1.000 |
|  | GMT [95% CI] | 598.78[527.79, 679.31] | 664.00[583.86, 755.14] | 0.279 |
| **Non-susceptible** |  |  |  |  |
| Anti-type I | n | 43 | 44 |  |
|  | Seroconversion, Rate% [95% CI] | 95.35[89.06, 100] | 84.09[73.28, 94.90] | 0.077 |
|  | GMT [95% CI] | 611.30[427.16, 874.81] | 604.44[385.05, 948.83] | 0.528 |
| Anti-type II | n | 20 | 25 |  |
|  | Seroconversion, Rate% [95% CI] | 95.00[75.13, 99.87] | 100[86.28, 100] | 0.444 |
|  | GMT [95% CI] | 204.36[131.02, 318.76] | 315.17[211.72, 469.17] | 0.379 |
| Anti-type III | n | 18 | 10 |  |
|  | Seroconversion, Rate% [95% CI] | 77.78[52.36, 93.59] | 100[69.15, 100] | 0.249 |
|  | GMT [95% CI] | 414.37[276.71, 620.54] | 699.39[382.72, 1278.07] | 0.209 |

**Supplementary Table 2** Seroconversion rates and GMCs 30 days after the 3rd dose in susceptible and non-susceptible infants between Group1 and Group3, PPS.

| **Serum antibodies** | **Parameters of immunogenicity** | **Group 1** | **Group 3** | ***P* value** |
| --- | --- | --- | --- | --- |
| **Susceptible** |  |  |  |  |
| anti-PT | n | 214 | 213 |  |
|  | Seroconversion, Rate% [95% CI] | 77.57[71.38, 82.97] | 84.51[78.94, 89.09] | 0.068 |
|  | GMC [95% CI] | 31.01[28.51, 33.73] | 39.26[36.16, 42.62] | <0.001 |
| anti-FHA | n | 214 | 208 |  |
|  | Seroconversion, Rate% [95% CI] | 80.84[74.92, 85.89] | 85.58[80.05, 90.05] | 0.194 |
|  | GMC [95% CI] | 29.37[27.64, 31.21] | 32.68[30.60, 34.89] | 0.019 |
| anti-D | n | 209 | 213 |  |
|  | Seroconversion, Rate% [95% CI] | 100[98.25, 100.00] | 100[98.28, 100] | 1.000 |
|  | GMC [95% CI] | 1.49[1.37, 1.63] | 1.61[1.48, 1.75] | 0.216 |
| anti-T | n | 204 | 212 |  |
|  | Seroconversion, Rate% [95% CI] | 100[98.21, 100] | 100[98.28, 100] | 1.000 |
|  | GMC [95% CI] | 4.73[4.38, 5.11] | 4.27[4.01, 4.55] | 0.042 |
| **Non-susceptible** |  |  |  |  |
| anti-PT | n | 1 | 4 |  |
|  | Seroconversion, Rate% [95% CI] | 0[0,97.50] | 0[0,60.24] | 1.000 |
|  | GMC [95% CI] | 42.30[-] | 42.91[19.66, 93.65] | 0.480 |
| anti-FHA | n | 1 | 9 |  |
|  | Seroconversion, Rate% [95% CI] | 0[0, 97.50] | 0[0, 33.63] | 1.000 |
|  | GMC [95% CI] | 38.00[-] | 43.20[33.17, 56.26] | 0.328 |
| anti-D | n | 6 | 4 |  |
|  | Seroconversion, Rate% [95% CI] | 83.33[35.88, 99.58] | 100[39.76, 100] | 1.000 |
|  | GMC [95% CI] | 1.68[0.82, 3.46] | 1.95[0.84, 4.55] | 0.770 |
| anti-T | n | 11 | 5 |  |
|  | Seroconversion, Rate% [95% CI] | 90.91[58.72, 99.77] | 100[47.82, 100] | 1.000 |
|  | GMC [95% CI] | 2.94[1.78, 4.85] | 4.10[3.71, 4.53] | 0.368 |
